# Acacetin as a Potential Protective Compound against Cardiovascular Diseases

**DOI:** 10.1155/2022/6265198

**Published:** 2022-03-02

**Authors:** Chunxuan Wu, Jianhua Yan, Wei Li

**Affiliations:** Department of Cardiology, Xinhua Hospital, School of Medicine, Shanghai Jiao Tong University, Shanghai, China

## Abstract

Acacetin (5,7-dihydroxy-4′-methoxyflavone) is the major bioactive component of the traditional Chinese medicine “Snow lotus”. As a natural flavonoid compound, it has been shown to have good pharmacological effects such as anti-inflammatory, anticancer, and anti-obesity. Among them, its prominent role in cardiovascular diseases (CVD) has received extensive attention from scholars in recent years. In this review, the protective effects of acacetin on a variety of cardiovascular diseases, as well as the existing problems and prospects, are discussed and summarized. This review also highlights the great potential of acacetin, a natural-derived Chinese medicine, as a cardiovascular agent candidate.

## 1. Introduction

Cardiovascular disease (CVD), including coronary heart disease, hypertension, myocardial infarction, and heart failure, is not only the main cause of death worldwide but also a major public health problem [1, 2]. Although many great achievements have been made in the prevention and treatment of CVD in the past decades, the morbidity and mortality of CVD continue to increase due to population growth and ageing [3]. Therefore, it is of great significance to develop alternative or complementary therapies for cardiovascular diseases.

Traditional Chinese medicine (TCM) has a history of more than 2,000 years. It is not only popular in China but also highly recognized in many countries in the world [4]. In recent years, more and more TCMs have been proved to be potential candidates for the treatment of cardiovascular disease, and their mechanisms is becoming clearer, such as berberine, curcumin, Wenxin Keli, resveratrol, and acacetin [5–8].

**Figure 1:**
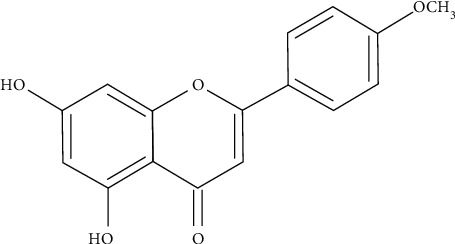
Chemical structure of acacetin.

**Figure 2:**
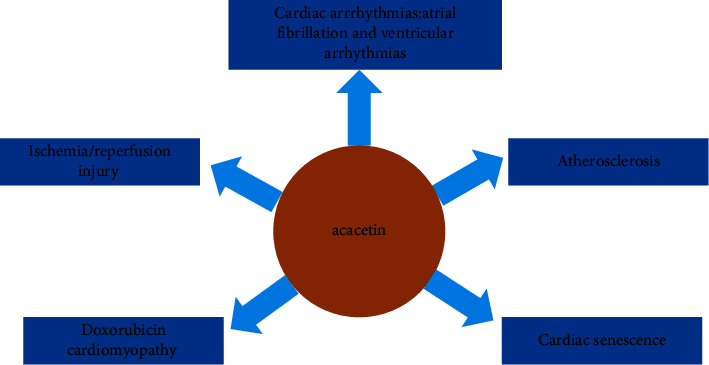
Pharmacological properties of acacetin in cardiovascular diseases.

Acacetin (5,7-dihydroxy-4′-methoxyflavone), one of the flavones of flavonoids, is a monomethoxy flavonoid, which exists in a variety of plants in free form or glycoside form, including *Robinia pseudoacacia*, *Chrysanthemum indicum*, and other species [9, 10] (Figure 1). Acacetin has attracted wide attention from the scientific community because of its various pharmacological activities, including anti-inflammatory, anticancer, anti-obesity, anti-diabetic, neuroprotective, and cardioprotective effects [9, 11]. Among them, the application prospect of acacetin in the CVD field is the most noteworthy (Figure 2). It has good curative effects on CVD with almost no toxic reactions, such as atrial fibrillation, ischemia/reperfusion injury, atherosclerosis, doxorubicin cardiomyopathy, and cardiac senescence.

This review focuses on the protective effects of acacetin on CVD, as well as the main signal pathways regulated under different pathophysiological conditions in CVD. This review also emphasizes the current problems and challenges related to acacetin in the treatment of CVD, which need further experimental exploration and make it a candidate for the prevention and treatment of CVD.

## 2. Pharmacological Effects on Cardiovascular Diseases

### 2.1. Cardiac Arrhythmias

#### 2.1.1. Atrial Fibrillation

Atrial fibrillation (AF) is one of the most common cardiac arrhythmias in clinical practice, and its incidence increases with age [12]. Complications of cardiac and extra-cardiac caused by AF significantly increase morbidity and mortality [13, 14]. In the past two decades, catheter ablation has become the mainstream strategy of rhythm control recommended by clinical guidelines, but antiarrhythmic drugs are also an indispensable part of controlling AF and its recurrence [15, 16]. The development of atrial-selective antiarrhythmic drugs is important for the treatment of AF, because if the drug acts on the ventricles, it may prolong the action potential duration (APD) of the ventricles and even cause the appearance of lethal ventricular arrhythmias such as torsade de pointes (Tdp) [17,18].

Recently, Li et al. have demonstrated that acacetin can preferentially inhibit *I*_Kur_ (ultra-rapidly activated delayed rectifier potassium current), *I*_to_ (transient outward potassium current), *I*_KACh_ (acetylcholine activated potassium current), and SKca (small conductance Ca^2+^-activated potassium channels current), while it had no effect on the Na^+^ current, L-type Ca^2+^ current, or inward-rectifier K^+^ current in guinea pig cardiac myocytes [19, 20]. Among them, *I*_kur_ is specifically expressed in the human atrium but not in the ventricle, which is an ideal target for atrial-selective antiarrhythmic drugs [21, 22]. In addition, previous studies have shown that selective *I*_KACh_ inhibitors prevent AF induced by vagal stimulation [23, 24]. Intraduodenal administration of acacetin can effectively prevent AF in anesthetized dogs by increasing the APD and prolonging the effective refractory period (ERP) in atrial myocytes. More importantly, compared with sotalol, acacetin can effectively prevent AF without QT interval (QTc) prolongation. These results suggest that acacetin is an atrium-selective agent and has great potential to treat AF. Considering that acacetin has poor water solubility, it can only be taken orally and cannot be used for intravenous injection to terminate AF. In 2016, a water-cooled phosphate acacetin prodrug which can be quickly converted into the active form acacetin was designed [25]. The study shows that the synthesized acacetin prodrug is highly water-soluble and safe. Furthermore, it can also effectively terminate experimental AF in beagle dogs.

The molecular determinants of acacetin's ability to block *I*_to_ and *I*_kur_ were further investigated [26, 27]. The results show that acacetin has a use-dependent and frequency-dependent blocking effect on hKv4.3 (coding *I*_to_) and hKv1.5 (coding *I*_kur_) channels. Acacetin, as an antagonist of the hKv4.3 channel, interacts with T366A and T367A located in the P-loop helix and V392A, I395A, and V399A located in the S6-segment [26]. In addition, acacetin, as a hKv1.5 channel antagonist, interacts with V505A, I508A, and V512A located in the S6 domain [27].

In addition, the combination of acacetin and sodium channel blockers has also been shown to be a promising strategy for the treatment of AF [28].

#### 2.1.2. Ventricular Arrhythmias

*J* wave syndromes, mainly including Brugada syndrome (BrS) and early repolarization syndrome (ERS), are characterized by an abnormal transition point between the terminal part of the QRS complex and the ST segment, that is, the *J* point in the electrocardiogram. It is well known that *J* wave syndromes are associated with serious ventricular tachycardia (VT), ventricular fibrillation (VF), and other life-threatening events [29, 30]. Implantable cardioverter defibrillator (ICD) is recommended as the preferred therapy for the prevention of sudden cardiac death in high-risk patients with *J* wave syndrome. But the drugs for the diseases are extremely limited. Quinidine, an Ito inhibitor, is one of the few drugs that are effective in treating *J* wave syndromes [31]. More recently, Di Diego et al. have shown that acacetin can noticeably improve the electrocardiogram and arrhythmia performance in both the BrS and ERS experimental models by reducing I_to_ density, action potential notch, and *J* wave area [32].

### 2.2. Ischemia/Reperfusion Injury and Myocardial Infarction

In 2014, Yang et al. for the first time demonstrated that acacetin in vitro can reduce the hypoxia/reoxygenation injury to the cardiac myocardium and has a protective effect on the myocardium by reducing lipid peroxidation and enhancing the antioxidant capacity [33]. In this study, acacetin can significantly reduce the content of malondialdehyde (MDA), which is usually used to evaluate the oxidative damage of cells in the medium during hypoxia/reoxygenation injury. Subsequently, an acacetin phosphate prodrug was applied to the in vivo experiments of ischemia/reperfusion injury, and the studies showed that acacetin inhibited the apoptosis of myocardial cells by preventing the reduction of antioxidants such as SOD-2 and thioredoxin and reducing the release of inflammatory cytokines such as TLR4, IL-6, and TNF*α*, thus playing a protective role on the myocardium after myocardial infarction (MI) [34]. Further studies have confirmed that the antioxidant, anti-inflammatory, and antiapoptotic effects of acacetin on cells are mediated by AMPK-mediated Nrf2 activation [35]. Interestingly, a recent study demonstrated that autophagy is involved in the process of alleviation of myocardial hypoxia/reoxygenation injury by acacetin; that is, acacetin enhanced autophagy through activating the PI3K/Akt/mTOR pathway [36].

Myocardial infarction is one of the most prominent cardiovascular diseases, contributing significantly to death. Cardiac remodeling is an important process after myocardial infarction, which is closely related to the prognosis of the disease. Recent experiments have shown that acacetin can inhibit myocardial remodeling such as myocardial hypertrophy and fibrosis, reduce myocardial infarct size, and enhance left ventricular function after myocardial infarction [37]. The protective effect of acacetin on cardiac remodeling after myocardial infarction was attributed to its inhibition of MAPK and PI3K/Akt signaling pathways, which play important roles in myocardial remodeling after myocardial infarction [38].

### 2.3. Atherosclerosis

Atherosclerosis used to be regarded as a chronic inflammatory disease related to dyslipidaemia, but more and more evidence shows that oxidative stress is also involved [39]. Considering the powerful antioxidant and anti-inflammatory ability of acacetin, Yao et al. studied the potential of acacetin's application in atherosclerosis [40]. On the one hand, acacetin enhances the antioxidant defense of cells through the MsrA-Nrf2/Keap1 pathway and significantly reduces apoptosis. On the other hand, it can accelerate lipid metabolism in the liver and circulation, and has anti-inflammatory effects. In fact, statins, the widely used antihyperlipidemia drugs, have been demonstrated to alleviate oxidative stress by regulating the redox system, which may further increase the possibility of clinical use of acacetin in preventing and treating atherosclerosis-related CVD [41].

### 2.4. Doxorubicin Cardiomyopathy

Doxorubicin is an anthracycline antibiotic, which is widely used as an anticancer drug in clinics [42]. However, the most significant and harmful side effect of doxorubicin is the increasing cardiovascular toxicity events, including proarrhythmia, hypotension, and congestive heart failure [43]. Dexrazoxane is the only drug to be approved by the FDA to deal with cardiomyopathy caused by doxorubicin, but it has been demonstrated to increase the risk of secondary malignancy [44]. It is worth noting that reactive oxygen species (ROS) is considered to be the most important mechanism of doxorubicin cardiomyopathy [45]. Acacetin has been shown to reduce apoptosis and ROS production and enhance endogenous antioxidants (SOD1, SOD2, and HO-1) via Sirt1-dependent activation of AMPK/Nrf2 signals, thus inhibiting myocardial toxicity and cardiac dysfunction caused by doxorubicin [46]. Therefore, acacetin may be a very promising candidate for the treatment of doxorubicin cardiomyopathy in the future.

### 2.5. Cardiac Senescence

Ageing is considered an independent risk factor for cardiovascular disease, and cardiac senescence is closely related to the occurrence and development of cardiomyopathy in the elderly [47]. Since population ageing is a current situation in many countries, relevant anticardiac ageing treatments are urgently needed. Among them, the change in mitochondrial metabolism is considered as an important mechanism of myocardial ageing [48]. Interestingly, acacetin can significantly improve the cardiac function in the D-galactose-induced cardiac senescence model, reduce senescence markers such as P53 and P21, increase autophagy protein, and increase mitochondrial autophagy via Sirt1-mediated activation of the Sirt6/AMPK signaling pathway [49]. This study suggests that acacetin may be a promising drug candidate for treating ageing-related cardiovascular disorders.

## 3. Problems and Future Prospects

Although acacetin is a safe and promising candidate for the treatment of CVD, there are still many problems to be resolved in the future. One of the most difficult problems is the poor solubility and rapid metabolism of acacetin in vivo, which will be a major obstacle to the clinical development and utilization of acacetin in the future [50]. In 2016, a study showed us a way to improve acacetin's water-solubility by designing prodrugs to treat AF [25]. This method of designing prodrugs to change the properties of the drug and overcome the shortcomings of the drug plays a role in the initial development of the drug and the various stages of preclinical trials [51, 52]. However, there are few studies on the development of acacetin prodrugs.

On the other hand, although the pathophysiological conditions of various CVDs are simulated in animals, there are few applications of uniform animal experimental models that are recognized around the world. The in-depth research of acacetin in this area in the future will be an important milestone in promoting preclinical experiments.

In addition, the clinical pharmacological information of acacetin, which is considered to be an important part of drugs, such as absorption, metabolism, and toxicity, is not sufficient at present [53, 54]. Therefore, all of these need to be further explored and improved upon, so as to confirm the safety and effectiveness of acacetin in human body. Overall, there is still a great distance to the routine clinical application of acacetin. However, it cannot be denied that acacetin is a promising natural candidate for cardiovascular drugs.

## 4. Conclusion

In general, this review emphasizes the great potential of acacetin as a traditional Chinese medicine on cardiovascular disease and summarizes the remarkable effects and related possible mechanisms of acacetin against atrial fibrillation, ischemia-reperfusion injury, atherosclerosis, doxorubicin cardiomyopathy, and cardiac senescence. Further studies are warranted to resolve the current problems of acacetin, such as poor solubility and rapid metabolism in vivo, which may help prevent and treat cardiovascular diseases in the future.

## Abbreviations

AF: Atrial fibrillation
APD: Action potential duration
BrS: Brugada
CVD: Cardiovascular diseases
ERP: Effective refractory period
ERS: Early repolarization syndromes
I_KACh_: Acetylcholine-activated potassium current
I_Kur_: Ultra-rapidly activated delayed rectifier potassium current
I_to_: Transient outward potassium current
MI: Myocardial infarction
QTc: QT interval
ROS: Reactive oxygen species
SKca: Small conductance Ca^2+^-activated potassium channels current
TCM: Traditional Chinese medicine
Tdp: Torsade de pointes
VT: Ventricular tachycardia
VF: Ventricular fibrillation.
